# Oral Health Status, Behaviours and Oral Healthcare Utilization among Indian Migrants Compared to the Host Population in the Netherlands: A Descriptive Cross-sectional Study

**DOI:** 10.1007/s10903-023-01553-7

**Published:** 2023-10-17

**Authors:** Amandeep Pabbla, Charles Agyemang, Geert van der Heijden, Denise Duijster

**Affiliations:** 1 Department of Oral Public Health, Academic Centre for Dentistry Amsterdam (ACTA), University of Amsterdam and VU University, Gustav Mahlerlaan 3004, Amsterdam, 1081 LA The Netherlands; 2 Department of Public Health, Academic Medical Centre (AMC), University of Amsterdam, Amsterdam, The Netherlands; 3 Division of Endocrinology, Diabetes, and Metabolism, Department of Medicine, Johns Hopkins University, Baltimore, MD USA

**Keywords:** Indian migrants, Oral health status, Oral health behaviours, Oral healthcare utilization

## Abstract

The aim of this study was to assess the oral health status, oral health behaviours and oral healthcare utilization among Indian migrants living in the Netherlands and how they compare with the host population. Based on a random sample from Dutch municipalities, cross-sectional data were obtained for the Indian migrants living in the Netherlands (n = 148) and the host population (n = 244). A questionnaire was used to collect information on socio-demographic, self-reported oral health status, oral health behaviours and oral healthcare utilization. The distribution of self-reported oral health variables for both groups were tabulated and compared using logistic, ordinal and multinomial regression analysis. When adjusted for covariates such as age, gender, marital status, education, income, occupation and dental insurance, regression analysis for oral health status showed that the odds of reporting oral impact on daily performances (OIDP) was 5.87 times higher for Indians compared to the host population (95%CI:3.45;9.65). In contrast, the odds of Indians reporting bleeding gums [OR = 0.44 (95%CI:0.27;0.73)] and diagnosed with gum diseases [OR = 0.23(95%CI:0.13;0.39)] were lower than the host population. Also, the odds of consuming alcohol and cakes or chocolates was significantly lower among Indian migrants compared to the host population [(OR = 0.15(95%CI:0.09;0.25)] and [OR = 0.33(95%CI:0.21;0.52)], respectively. But the odds of consuming sugar in hot beverages were significantly higher among Indians [OR = 10.44(95%CI:5.99;18.19)]. The odds of Indians visiting a dental professional were 9.22 times (95%CI:4.62;18.40) lower compared to the host population. We found that oral health status and behaviours among Indian migrants were different in certain aspects compared to the host population. However, their oral healthcare utilization remained overall lower. The underlying determinants for such observations merit further research. Migrant friendly approach from both the dental professionals and policy makers can encourage dental visits and improve the utilization patterns among Indians migrants in the future.

## Introduction

The Global Burden of Disease Study 2019 estimated that oral diseases affect close to 3.5 billion people worldwide [1]. Also, oral diseases disproportionally affect socially disadvantaged people [2], especially the vulnerable groups in society, including ethnic minority groups and migrants as they experience additional risks to their oral health [2, 3].

In the host country, migrants not only face challenges such as legal status, housing, education and employment [4], they also undergo varying degrees of social pressures and stressors relating to social and economic inequalities, language and different cultural norms. Migrants not only carry the burden of oral diseases, but they also bring forth their own cultural beliefs, norms and traditions with respect to (oral) health and healthcare, most of these vary considerably from the host society [4, 5]. In addition, understanding and navigating the infrastructure and organisation required to access the (oral) healthcare in the host country adds up to their difficulties. At group level this may lead to an increased risk of a poorer oral health among migrants compared to the host population.

Several publications report on the presence of oral health inequalities, varied oral health behaviours and differences in oral healthcare utilization among migrants. A recent systematic review found a higher prevalence of dental caries among migrant adolescents compared to the host population in Germany and Spain [6]. In contrast, studies from the United Kingdom (UK), Denmark and Sweden showed a lower prevalence of dental caries among migrants compared to the host population [6]. Similarly, for oral health behaviours, a cross sectional dental health survey in the UK reported that migrants with Asian and African background were more likely to add sugar to hot drinks but were significantly less likely to consume sweets and cakes compared to White British [7]. Furthermore, studies report that oral healthcare utilization is generally lower among the migrant population, which was associated with inadequate proficiency in the language skills [8], old age [9] and gender [10]. But so far there is no consistent pattern of factors reported to be associated with lower oral healthcare utilization.

This may relate to the heterogeneous study of migrant populations, resulting in discrepant findings on oral health status, oral health practices and beliefs and dental visits. Therefore, for a better understanding of the relationship between migration and (oral) health, it is important to concentrate on one clearly defined and delineated group of migrants and to compare them with either the host population or to their native population in their home country [6, 11, 12].

For this study, we consequently focused on the oral health status, behaviours and oral healthcare utilization among one target migrant group, the Indian migrants living in the Netherlands, who share common demographic characteristics and share similar cultural and social values pertaining to (oral) healthcare practices and dental visits. Currently, an estimated 58,460 Indians (exclusive of Surinamese Hindustanis) live in the Netherlands [13], making them one of the largest minority groups in the Netherlands. A recent systematic review and meta-analysis showed the overall prevalence of the periodontal disease among Indians is 51%, which means that about 320 million Indians have some form of periodontal disease [14]. Although studies on oral health among South Asians have suggested that Indian migrants have generally better oral health compared to other ethnic groups [7], the research is sparce and mostly limited to the UK and almost non-existent in other European countries.

The aim of our study was therefore to assess the oral health status, including assessment of oral impact on daily performances (OIDP), oral health behaviours and oral healthcare utilization among Indian migrants living in the Netherlands and how they compare with the host population. This study is a part of a broader research project investigating the impact of migration on oral health outcomes using Indian migrants living in the Netherlands, which includes a systematic review and a qualitative research on the factors associated with oral healthcare among Indian migrants. The findings of this research project could provide valuable insights into the oral health needs and challenges faced by Indian migrants in the Netherlands, contribute to improving cultural competency among oral healthcare providers and highlight barriers to accessing oral healthcare services among them.

## Methods

For this descriptive comparative study, we followed a cross-sectional survey design. We gathered data on the Indian migrants and the host population via questionnaires. The Medical Ethics Review Board of the Medical Centre of the VU University Amsterdam approved the project (reference number 2020.479).

### Study Population and Sampling

The study population consisted of two groups. As the inclusion criteria, the Indian adult migrants aged 18 years and above, born in India and living in the Netherlands for at least five years were included. This ensured that these Indian migrants had already entered the Dutch healthcare system. For the host population, the inclusion criteria were those aged 18 years and above and born and living in The Netherlands. The study groups are based on random sampling from the inhabitants registered to live in Dutch municipalities. The Rijksdienst voor Identiteitsgegevens (RvIG) authorised the Central Bureau of Statistics, Netherlands (CBS) to draw a random sample from the registry of Dutch Municipalities (Basis Registratie Personen, BRP).

Indian migrants living in the Netherlands are mostly concentrated in five major cities, namely: Amsterdam, including Amstelveen, Utrecht, Rotterdam, The Hague and Eindhoven. The sample size was based on the number of natural teeth as a parameter for oral health. To explore differences in the number of natural teeth between groups, a power calculation indicated that a sample of 730 individuals was required. The calculation was based on the upon detecting a minimum difference of two natural teeth (effect size = 0.21) using the following parameters: 80% of power, 5% level of significance and a standard deviation of 9.4 natural teeth in Dutch adults (host population) aged 25–75. To the present sample size (n = 730), 20% excess was added due to multivariate analyses and 15% excess was added to allow for missing data. After this, we got the inflated sample size of 1007. We then multiplied this inflated sample number by 2 to compensate for 50% response rate and we got the sample size of 2014 (1007 Indians and 1007 host population). We finally increased the sample size based on the concentration of Indian migrants in the five cities mentioned above and got the final sample of 600 participants (300 Indian migrants and 300 host population) from each city, making a total of 1500 (300 × 5) Indian migrants and 1500 (300 × 5) host population, giving us our sample size to be 3000.

### Data Collection and Processing

Between February 2021 and April 2021, all potential participants received an information letter for study participation at their postal addresses. The survey questionnaire included a written consent form with space for signature as the front page. In addition, a return envelope was attached as well. The letter included a link for online submission of the questionnaire for those preferring digital completion. For this we used Qualtrics Online Survey Software (version February 2021). We sent a first reminder letter after three weeks, and a second and final reminder two weeks thereafter including another copy of the questionnaire. We used a questionnaire in Dutch for the host population and a questionnaire in English for the Indian migrants. For those preferring online completion, the questionnaire was available in English, Dutch, and also Hindi for the Indian migrants.

### Variables Recorded

Self-rated oral health was measured using the question: ‘How would you rate your oral health?’, with ordinal response categories grouped as: very good, good, fair to very poor, which was then regrouped as good versus bad, for analysis [15]. We also collected data on bleeding gums in the past three months, diagnosed gum disease, toothache in the past three months, loose teeth and use of a denture. The responses for these were dichotomised as yes or no. In addition, oral impact on daily performances (OIDP) scale was used as an oral health related quality of life measure, with responses on Likert scale (0 = no effect to 5 = very high effect) [16]. We used the sum score, and we dichotomised responses into yes (1 or more impacts) or no (zero impact).

Variables related to oral health behaviours included questions on habits, such as smoking (yes or no) and alcohol consumption (never, several times per month, or several times per week). For frequency of sugar consumption in the form of cakes and chocolates, fizzy drinks, and addition of sugar in hot beverages, the responses were never, several times per month, or several times per week. We also asked questions on oral hygiene as ‘How do you clean your teeth?’ with manual toothbrush, electric toothbrush or both as response options and use of fluoride in toothpaste, with dichotomised response as yes or no.

Oral healthcare utilization was assessed with the questions on visiting the dental professional within the last 12 months/ before COVID and whom did they visit (no visits, visited only the dentist, visited only the dental hygienist or visited both). In addition, we asked ‘How satisfied are you with the dental care provided to you in the Netherlands?’, with responses on a Likert scale: satisfied or neutral or unsatisfied. Information on relevant socio-demographic characteristics, notably age, gender, country of birth, marital status, education, occupation, income and dental insurance was also recorded.

### Data Analysis

We used descriptive statistics to report the frequency distribution of variables in the two groups. For all categorical variables, we used the chi-square test to compare proportional differences between Indian migrants and the host population. Only age was used as a continuous variable and Mann Whitney U-test was used for comparisons as it was not normally distributed.

We used univariate regression analysis (logistic regression for binary outcomes, ordinal regression for rank and responses on a Likert scale and multinomial regression for nominal outcomes with more than two categories) to explore and describe the between group differences for all oral health outcome variables.

For each variable in the equation, the following statistics were calculated: estimated odds ratio (exp[B]) and confidence intervals (CI). Thereafter, we used multivariable regression analyses to assess the association of the two groups (Indians and the host population) with all oral health outcomes: oral health status, behaviours and oral healthcare utilization. This was done while adjusting for the selected covariables such as age, gender, marital status, education level, occupation and income. Data from questionnaires were processed for analysis using IBM SPSS Statistics for Windows, version 27.0 (IBM Corp., Armonk, N.Y., USA). A p-value of < 0.05 was used as an arbitrary cut-point for statistical significance.

## Results

We received the postal addresses of a stratified sample of 2,885 potential participants (1,498 host population and 1,387 Indian migrants) from CBS. Two hundred and fifteen envelops with the invitation were sent back since people had moved houses. A total of 392 people responded: 244 host population and 148 Indian migrants, giving a total response rate of 13.5%. In Table 1, sociodemographic characteristics of both the groups are reported. The proportion of Indian migrants with higher educational level and highly paid jobs was higher compared to the host population.

**Table 1:** Basic demographic characteristics of both groups: Host population and Indian migrants

	Host population	Indian migrants
Total (n)	244	148
^a^Age (years)	Median (Q1-Q3)	Median (Q1-Q3)
	43 (29–56)	36 (32–43)
	n (%)	n (%)
Gender		
Males	98 (40)	95 (64)
Females	146 (60)	53 (36)
Marital Status		
Married /registered partnership	159 (65)	117 (79)
Single/ divorced	85 (35)	31 (21)
Education		
Low to medium	48 (20)	5 (3)
High	196 (80)	141 (97)
Income		
€0–1800/month	36 (15)	7 (5)
€1800–2600/month	26 (11)	12 (8)
€2600–4000/month	74 (32)	28 (19)
>€4000/month	99 (42)	100 (68)
Dental Insurance		
Yes	126 (52)	76 (51)
No/ I do not know	115 (48)	72 (49)
Occupation		
Unemployed, unable to work	45 (19)	15 (10)
Paid worker	193 (81)	133 (90)

^a^Age was used as a continuous variable and was not normally distributed

Missing: Age (n = 3), education (n = 2), income (n = 10), dental insurance (n = 3), occupation (n = 6)

Table 2 shows the frequency distribution and regression analysis of the oral health status among the two groups. The odds of Indians rating their oral health as fair to poor was 1.91(95% CI:1.15;3.20) times higher than that of the host population The odds of Indians to report OIDP was 5.77(3.45;9.65) times higher than the host population However, the odds of Indians to report bleeding gums and to report being diagnosed with gum diseases was significantly lower than the host population [OR = 0.44(0.27;0.73) and OR = 0.23(0.13;0.39) respectively].

**Table 2:** Frequency distribution and the Binary regression analysis on oral health status for both groups: Host population and Indian migrants

	Descriptive analysis				Regression analysis	
	n (%)			n (%)	Crude) ^a^ OR [95% CI]	(Adjusted) ^b^ OR [95% CI]
Self-rated oral health						
	*Very good to good (ref)*			*Fair to poor*		
Host population	173 (71)			70 (29)	Ref	Ref
Indian migrants	88 (60)			60 (40)	1.68 [1.10; 2.59]	1.91 [1.15; 3.20]
OIDP (oral impact on daily performances) scale						
	*No impact (ref)*			*Yes impact*		
Host population	159 (66)			80 (34)	Ref	Ref
Indian migrants	38 (26)			110(74)	5.75 [3.64; 9.08]	5.77 [3.45; 9.65]
Bleeding gums in the past 3 months						
	*No (ref)*			*Yes*		
Host population	137 (56)			105 (73)	Ref	Ref
Indian migrants	107 (44)			39 (27)	0.46 [0.29; 0.71]	0.44 [0.27; 0.73]
Diagnosed with gum diseases						
	*No (ref)*			*Yes*		
Host population	119 (49)			125 (51)	Ref	Ref
Indian migrants	120 (81)			28 (19)	0.22[0.14; 0.36]	0.23 [0.13; 0.39]
Toothache in the past 3 months						
	*No (ref)*		*Yes*			
Host population	203 (83)		121 (82)		Ref	Ref
Indian migrants	41 (17)		27 (18)		1.10 [0.65;1,89]	0.93 [0.50; 1.73]
Loose teeth in the mouth						
	*No (ref)*	*Yes*				
Host population	235 (96)	9 (4)			Ref	Ref
Indian migrants	133 (90)	15 (10)			2.94 [1.26; 6.91]	4.40 [1.57; 12.29]
Use a denture						
	*No (ref)*			*Yes*		
Host population	229 (94)			15 (6)	Ref	Ref
Indian migrants	131 (89)			17 (12)	1.98 [0.96; 4.10]	3.11 [1.21;7.99]

^a^Regression analysis when adjusted for country of birth

^b^Adjusted for age, gender, marital status, education, income, occupation and dental insurance

Table 3 shows the frequency distribution and the regression analysis of the oral health behaviours among the two groups. The odds of consuming alcohol and cakes or chocolates was significantly lower among Indian migrants compared to the host population [OR = 0.15(0.09;0.25) and OR = 0.33(0.21;0.52), respectively] after full adjustments. In contrast, the odds of Indians consuming sugar in hot beverages were significantly higher compared to the host population [OR = 10.44(5.99;18.19)]. For oral hygiene practices in the adjusted model, the odds of Indians using manual toothbrushes against using of both manual and electric toothbrushes was higher [OR = 2.93(1.48;5.80)] than the host population. Also, the odds of Indians using fluoridated toothpaste was 2.5(1.51;4.14) times lower than the host population. No significant association was seen between other variables for oral health behaviours in the two groups.

**Table 3:** Frequency distribution and the regression analysis on oral health behaviour for both groups: Host population and Indian migrants

	Descriptive analysis						Regression analysis			
	n (%)		n (%)				Crude) ^a^ OR [95% CI]		(Adjusted) ^b^ OR [95% CI]	
Smoking [Binary logistic regression] No (*ref*)										
	*No*		*Yes*							
Host population	223 (91)		21 (9)				Ref		Ref	
Indian migrants	135 (91)		13 (9)				1.02 [0.50; 2.11]		1.37 [0.60; 3.17]	
Alcohol consumption [Ordinal logistic regression] Never (*ref*)										
	*Never*	*Monthly*				*Weekly*				
Host population	17 (7)	113 (46)				114 (47)	Ref		Ref	
Indian migrants	35 (24)	100 (68)				13 (9)	0.16 [0.10; 0.25]		0.15 [0.09; 0.25]	
Cakes, chocolates [Ordinal logistic regression] Monthly (*ref*)										
	*Monthly*	*Weekly*				*Daily*				
Host population	100 (41)	71 (30)				73 (30)	Ref		Ref	
Indian migrants	97 (66)	44 (30)				7 (5)	0.30 [0.20; 0.46]		0.33 [0.21; 0.52]	
Fizzy drinks [Ordinal logistic regression] Never (*ref*)										
	*Never*	*Monthly*				*Weekly*				
Host population	101 (41)	88 (36)				55 (23)	Ref		Ref	
Indian migrants	63 (43)	69 (47)				16 (11)	0.77 [0.52; 1.19]		0.74 [0.48; 1.14]	
Sugar intake-sugar in hot drinks [Binary logistic regression] No (*ref*)										
	*No*			*Yes*						
Host population	202 (83)			42 (17)			Ref		Ref	
Indian migrants	50 (34)			98 (66)			9.43 [5.86; 15.17]		10.44 [5.99; 18.19]	
Cleaning teeth [Multinomial regression] Use both types (*ref*)										
	*Manual toothbrush*	*Electric toothbrush*			*Both types*					
Host population	158 (24)	131 (54)			54 (22)		Ref		Ref	
Indian migrants	73 (50)	50 (34)			23 (16)		Manual vs. Both: 3.00 [1.63; 5.37]	Electric vs. Both: 0.90 [0.50; 1.61	Manual vs. Both: 2.93 [1.48; 5.80]	Electric vs. Both: 0.86 [0.45; 1.67]
Fluoridated toothpaste [Binary logistic regression] Yes (*ref*)										
	*Yes*		*No*							
Host population	183 (75)		61 (25)				Ref		Ref	
Indian migrants	85 (58)		62 (42)				2.19 [1.41; 3.39]		2.50 [1.51; 4.14]	

^a^Regression analysis when adjusted for country of birth

^b^Adjusted for age, gender, marital status, education, income, occupation and dental insurance

Table 4 shows the frequency distribution and the regression analysis of the oral healthcare utilization among the two groups. In an adjusted model, the odds of Indians visiting no dental professional in the past 12 months were 9.22(4.62;18.40) times higher compared to visiting both, a dentist and a hygienist. For satisfaction with dental care, the odds of Indians to report being unsatisfied were 4.90(2.58;9.22) times higher compared to the host population. Apart from regression analysis, we also report the frequency distribution of additional aids used for keeping the teeth clean, reasons given for visiting a dental professional and the kind of treatments received among the two groups. (appendix Table 1a, 1b).

**Table 4:** Frequency distribution and the regression analysis on oral healthcare utilization for both groups: Host population and Indian migrants

	Descriptive analysis			Regression analysis			
	n (%)	n (%)	n (%)	(Crude) ^a^ OR [95% CI]		(Adjusted) ^b^ OR [95% CI]	
Visit dental professional [multinomial logistic regression] Both dentist and hygienist (*ref*)							
	*Both (dentist and hygienist)*	*Dentist*	*None*				
Host population	146 (60)	82 (34)	15 (6)	Ref		Ref	
Indian migrants	50 (35)	28 (20)	65 (45)	Dentist Vs Both: 1.00 [0.58; 1.70]	None Vs Both: 12.65 [6.63; 24,16]	Dentist Vs Both: 1.17 [0.65; 2.11]	None Vs Both: 9.22 [4.62; 18.40]
Satisfaction with dental care [ordinal logistic regression] Satisfied (*ref*)							
	*Satisfied*	*Neutral*	*Unsatisfied*				
Host population	213 (90)	21 (9)	2 (1)	Ref		Ref	
Indian migrants	81 (62)	42 (32)	8 (6)	5.76 [3.31; 10.04]		4.90 [2.58; 9.22]	

^a^Regression analysis when adjusted for country of birth

^b^Adjusted for age, gender, marital status, education, income, occupation and dental insurance

## Discussion

In this study we found that Indians reported their oral health to be poorer with high OIDP impact in comparison to the host population. Yet they reported lower bleeding gums and gum diseases compared to the host population. Indian migrants reported lower frequency in consuming alcohol, cakes and chocolates but higher consumption of sugar in their hot beverages. Also, use of manual toothbrushes was higher, but use of fluoridated toothpastes was lower among Indians than among the host population. Compared to the host population, oral healthcare utilization was lower among Indians as was their satisfaction with the dental healthcare professionals.

Among the oral health status, we found that OIDP impact was higher among the Indians as compared to the host population. Although we could not find other studies assessing the OIDP among Indian migrants, Arora et al. [7] reported contrasting results where Indians were less likely to report difficulties in eating due to dental problems compared to other minority groups in the United Kingdom, UK. On the other hand, two studies by Newton et al. [17, 18] found that when using subjective oral health status indicators, Indian migrants reported higher impact on the activities of daily life due to oral symptoms compared to other ethnic groups. Cultural variation in perceived oral health status could explain the differences in the concept of oral health across various ethnic groups.

Furthermore, in our study, Indians reported lower bleeding gums and were less likely to be diagnosed with gum diseases compared to the host population. For the first time in 1999, Newton et al. [19] published an article, which suggested better oral health among ethnic groups, including Indians compared to the data from the Adult Dental Health Survey, UK. Since then, many studies have found comparable results or have completely refuted it. For instance, a study in Singapore reported worse periodontal health among Indians compared to other ethnic groups [20]. Periodontal problems are relatively more prevalent among aging population. As Indian migrants in our study were younger, they may not have started with any form of periodontal issues. Also, bleeding gums being the early indicator of periodontal problems, are usually missed or dismissed by most unless pointed out by the dental healthcare professionals. Since we observed lower dental visits among Indians, this could have led to under reporting of gum diseases. Building on existing oral health status using longitudinal or qualitative research designs may help to identify and discuss plausible explanation for such findings in future.

Frequency of alcohol consumption among Indian migrants was lower compared to the host population. For most Indians, consumption of alcohol is culturally related rather than just a social norm. Vora et al. [21] also observed that alcohol consumption among Indians was less frequent compared to other migrant groups. We also found that Indians were less likely to consume cakes and chocolates but add more sugar in their hot drinks compared to the host population which is similar to what Arora et al. [7] also observed in their study. Sweets are integral to Indian traditions, especially the Indian festivals where consumption of Indian sweets is high, but not in the forms of cakes and chocolates. Also, for most Indians, traditional Indian tea needs to be a blend of milk and sugar as well. This would explain their high consumption of sugar in hot beverages. Oral hygiene practices among Indians showed lower use of fluoridated toothpaste which could be due to unawareness as a result of their lower dental visits or due to the use of herbal based toothpastes that are very popular among Indians [22] and are available at Indian stores across Netherlands.

It was also interesting to observe that Indian migrants had lower dental visits despite 68% being in a high-income bracket, 97% being well-educated, and more than 51% having dental insurance. This could be explained by the difference in the social norms between Netherlands and India. From the initial school days, most of the host population is exposed to routine dental visits to a hygienist as well as a dentist. Whereas dental visits in India are essentially associated with some dental problem. In addition, Arora et al. [7] also highlighted the barrier presented by Indian and Pakistani participants in the UK while visiting a dental professional. These groups felt that their inability to explain their dental problems might prolong their dental treatment, thereby increasing treatment costs. Similarly, migrants in the Netherlands also have access to the same healthcare services as Dutch citizens, including oral healthcare. However, there might be certain challenges faced by migrants, including language barriers, cultural differences in understanding oral health practices, and potentially unfamiliarity with the Dutch healthcare system [23].

Our study presents certain strengths and limitations. We aimed to collect oral health data only among Indian migrant population as they share similar cultural background, making our target population as homogenous as possible. We used stratified random sampling method for recruitment of study subjects, rather than rely on convenience sampling technique, which has been pointed out as a limitation in migrant oral health research [6]. In addition, Indian migrants included in this study differ from other vulnerable groups such as refugees and asylum seekers, not only in demographic characteristics, but their vulnerability is also attributed to political unrest, violence or psychological and physical injuries [24]. Hence, policies and activities related to their oral health differ considerably from those migrants who are already a part of the host healthcare system [24]. Since this was a questionnaire-based survey, only those with ability to read and write in English, Dutch or Hindi could participate. In addition, the online version of the questionnaire could only work for people with some form of digital know-how. We treat this as the strength of our study because this enabled participation of people with similar literary skills in both the groups. This could have resulted in reducing discrepancies in demographic characteristics across the groups.

The response rate in this study was low, only 13.5%, which could have resulted in selection bias and lower representation of the target groups, hence the findings need to be viewed with caution. Literature makes suggestions to opt for additional strategies to improve representation among migrant population, ranging from direct recruitment like door-to-door collection of data to indirect methods like community partnership [25]. Since this research was conducted during the COVID lockdown period, we could only contact participants via their postal addresses given to us by CBS. Other means of getting in touch with the Indian community via social media, community leaders, events and festival meetings were not possible during this time. In addition, COVID also had an impact on the self-reported oral health needs of people in general, which could similarly have influenced our study population as well [26, 27]. Also, this paper is cross sectional in design and hence there are no temporal relationships established. Although most systematic research on migrant oral health have stressed the need for qualitative and longitudinal studies [6, 11], the choice of descriptive design stems from the lack of research on oral health among one migrant group with similar background attributes. Also, we may have encountered certain bias, such as use of questionnaires for self-reported oral health status may have led to recall bias [28]. Socio demographic comparisons of our sample showed most Indian migrants were from higher education and higher socioeconomic background, resulting in selection bias. Hence the results of our findings need to be interpreted with caution.

In conclusion, we found that although Indian migrants were more likely to rate their oral health as poor, yet they reported lower gum diseases than the host population. Similarly, Indians had high consumption of sugar, but the forms differed: not as cakes and chocolates but in hot beverages. However, the oral healthcare utilization among Indian migrants remained consistently lower than the host population. These observations highlight the need for further research to gauge a deeper understanding of the differences in oral health among Indian migrants and the host population. In addition, determinants that play a vital role in influencing the oral health among Indian migrants can be built upon such cross sectional data. Furthermore, when treating patients with migrant backgrounds, a more culturally sensitive approach from oral health professionals can encourage dental visits among them and help identify barriers for underutilization. Since the Dutch healthcare policies emphasis on public health and prevention, policy makers could consider allocating resources for targeted oral health promotion campaigns, focusing on promoting good oral hygiene habits, healthy diets, and regular dental check-ups. for vulnerable groups, such as Indian migrants.

## Data Availability

The data that support the findings of this study are available on request from the corresponding author. The data are not publicly available due to privacy restrictions.
